# Application of Single-Particle Mass Spectrometer to Obtain Chemical Signatures of Various Combustion Aerosols

**DOI:** 10.3390/ijerph182111580

**Published:** 2021-11-04

**Authors:** Hee-joo Cho, Joonwoo Kim, Nohhyeon Kwak, Heesung Kwak, Taewan Son, Donggeun Lee, Kihong Park

**Affiliations:** 1 School of Earth Sciences and Environmental Engineering, Gwangju Institute of Science and Technology, 123 Cheomdangwagiro, Buk-Gu, Gwangju 61005, Korea; cheejoo@kier.re.kr (H.-j.C.); joonwookim@gm.gist.ac.kr (J.K.); kwak0087@umn.edu (N.K.); dbechkam@ktl.re.kr (H.K.); 2 School of Mechanical Engineering, Pusan National University, 2, Busandaehak-ro 63beon-gil, Geumjeong-gu, Busan 46288, Korea; jjoopopo@naver.com (T.S.); donglee@pusan.ac.kr (D.L.)

**Keywords:** single-particle mass spectrometer, combustion aerosols, chemical signature, laser ionization

## Abstract

A single-particle mass spectrometer (SPMS) with laser ionization was constructed to determine the chemical composition of single particles in real time. The technique was evaluated using various polystyrene latex particles with different sizes (125 nm, 300 nm, 700 nm, and 1000 nm); NaCl, KCl, MgCO_3_, CaCO_3_, and Al_2_O_3_ particles with different chemical compositions; an internal mixture of NaCl and KCl; and an internal mixture of NaCl, KCl, and MgCl_2_ with different mixing states. The results show that the SPMS can be useful for the determination of chemical characteristics and mixing states of single particles in real time. The SPMS was then applied to obtain the chemical signatures of various combustion aerosols (diesel engine exhaust, biomass burning (rice straw), coal burning, and cooking (pork)) based on their single-particle mass spectra. Elemental carbon (EC)-rich and EC-organic carbon (OC) particles were the predominant particle types identified in diesel engine exhaust, while K-rich and EC-OC-K particles were observed among rice straw burning emissions. Only one particle type (ash-rich particles) was detected among coal burning emissions. EC-rich and EC-OC particles were observed among pork burning particles. The single-particle mass spectra of the EC or OC types of particles differed among various combustion sources. The observed chemical signatures could be useful for rapidly identifying sources of atmospheric fine particles. In addition, the detected chemical signatures of the fine particles may be used to estimate their toxicity and to better understand their effects on human health.

## 1. Introduction

Atmospheric aerosols have received increased interest due to their adverse impacts on human health and climate change [1,2,3,4]. These aerosols can be emitted directly from various sources, such as biomass burning, vehicles, industry, power plants, deserts, and oceans (primary aerosols), and produced by a gas–particle conversion process in the ambient atmosphere (secondary aerosols). Their effects on human health and climate change are related to the physical and chemical properties of the particles. Once released into the air, they also undergo physical and chemical transformations (i.e., aging process), resulting in changes in particle size, morphology, and chemical composition [1,5].

Much effort has been focused on developing new instruments to measure the chemical composition of aerosols in real time [6,7,8]. Various versions of mass spectrometric techniques are available depending on the types of aerosol inlet, particle sizing, ionization, and ion detection [9,10,11]. A series of aerosol mass spectrometers (AMSs) (Aerodyne Inc, Chicago, IL, USA) employing thermal vaporization followed by electron impact (EI) ionization have been developed to provide quantitative and qualitative information on the chemical composition of fine particles in real time [8,12]. However, it is difficult to detect refractory materials such as black carbon (BC) and heavy metals using an AMS. A recent version of the AMS, known as the soot particle aerosol mass spectrometer (SP-AMS), is able to measure BC [13,14]. However, it is difficult to gain a clear picture of the elemental composition and mixing state of individual single particles that mainly consist of refractory materials.

By using a single-particle mass spectrometer (SPMS) technique based on laser ablation and ionization, the chemical composition of individual single particles consisting of refractory materials and their mixing states can be determined in real time [9,10,15,16,17,18,19,20,21]. The particle detection efficiency of the SPMS can be improved by using a laser triggering system, which consists of two light-scattering lasers, enabling the accurate estimation of the arrival time of particles at the ionization laser location [16,17,18,22,23,24,25,26]. However, since this kind of SPMS uses light scattered by particles, it is hard to detect nanoparticles or fine particles that scatter little light. An SPMS without a triggering system (i.e., free-firing mode) is able to detect nanoparticles or fine particles but sacrifices particle detection efficiency [15].

To locate and identify sources of atmospheric fine particles, it is essential to have the chemical profiles or signatures of fine particles produced from major candidate sources (database of chemical signatures) to compare with those of unknown particles in the ambient atmosphere. Since the SPMS is used to identify sources of atmospheric fine particles, chemical signatures of fine particles measured with the SPMS should be required. Furthermore, the detected chemical signatures of fine particles can be used to estimate their toxicity, which can be useful for understanding their effects on human health.

In this study, to better understand the chemical characteristics and mixing states of single particles in real time, a prototype SPMS employing laser ionization was constructed. Firstly, the SPMS was evaluated by using diverse particles with different sizes, chemical compositions, and mixing states. Then, the SPMS was applied to various combustion aerosols, and their chemical signatures and mixing states were determined, which can be useful for identifying sources of atmospheric fine particles and for better understanding their effects on human health.

## 2. Materials and Methods

**Figure 1:**
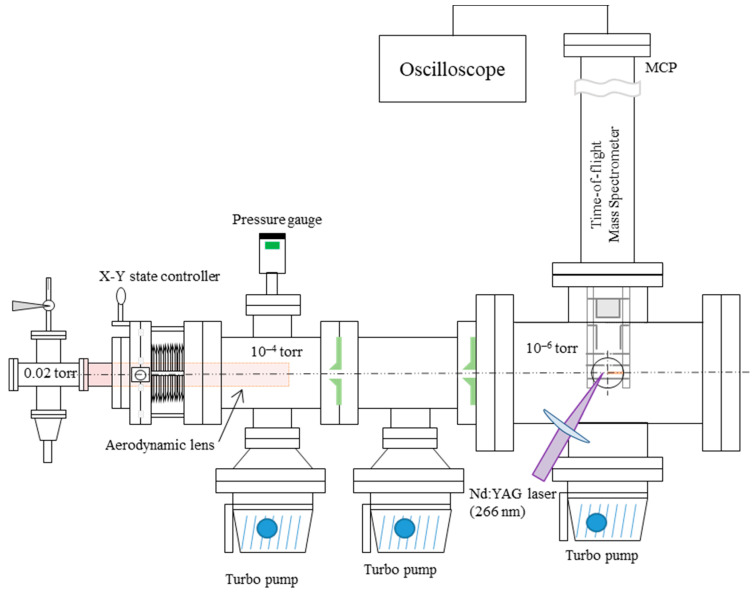
A schematic of the single-particle mass spectrometer.

The SPMS design is based on our previous works [8,15,27]. As shown in Figure 1, it mainly consists of an aerosol inlet system with an aerodynamic lens, a laser desorption/ionization system, and a linear time-of-flight mass spectrometer (TOF-MS). Three high vacuum chambers that are separated by a skimmer with a 3 mm inner diameter are maintained by turbo-molecular pumps (Navigator V301, Varian, Palo Alto, CA, USA). The pressure in the ionization chamber is kept below 10^−6^ Torr.

Aerosols are introduced through an inlet orifice with an inner diameter of 100 μm and a flow rate of 0.07 L/m, and, then, particles are focused into a particle beam of approximately 1 mm diameter by passing them through the aerodynamic lens system [28,29]. The aerodynamic lens is used as an interface to introduce aerosols from the atmosphere to the high vacuum system, and a collimated particle beam is generated for successful detection by the ionization laser. After exiting the aerodynamic lens, the particles achieve terminal velocity distribution, which is a function of their aerodynamic diameters during supersonic expansion [30,31,32]. A prototype aerodynamic lens is constructed, consisting of 12 sequential coaxial cylindrical orifices with varying diameters of 16, 7, 6, 5, 4, 5, 1.5, 14, 8, 5, 4.5, and 2.72 mm at the exit nozzle. The distance between the orifices is 40 mm. The particle beam diameter is much smaller than the diameters of the nozzle or skimmer. This can focus the particles onto the centerline of the orifices. The aerodynamic lens is mounted on the X-Y manipulator, which is used to adjust the direction and position of the particle beam, maximizing the transmission of particles to the laser ionization position.

A Nd: YAG laser (Ultra50, Quantel, Bozeman, MT, USA) with a 266 nm wavelength is used for the laser ionization of particles. The pulse energy of the laser beam is ~4 mJ/pulse before focusing the particles, and the calculated power density at the laser focal point is 1.3 × 10^5^ W/cm^2^. The maximum frequency of the laser is 20 Hz, and its pulse width is 3 ns. The frequency of TOF measurements is the same as the laser frequency. The measured mass range (*m/z*) is 0–230. When the single particle arrives at the center of the ionization point at the same time that the ionization laser fires (i.e., free-firing laser), the particle can be ionized to form ions by a multiphoton ionization process. The ions produced by laser ionization of the single particle are analyzed by the TOF-MS (RM Jordan Co., Grass Valley, CA, USA) in positive mode, and data are obtained by an oscilloscope (Waverunner 640Zi, Teledyne Lecroy, Ramapo, NY, USA) at a sampling speed of 1 GS/s. The ionization laser light detected by the photodiode is the triggering source of the TOF-MS. The ions produced from the particles are extracted from the drift tube to an electric-field-free region. The voltages are 4000 V for the repeller grid plate, 2850 V for the extraction grid plate, and 0 V for the acceleration grid plate and four steering plates in the TOF tube. After traveling along the 1 m long linear tube, the ions are detected by an MCP detector assembly (−3600 V), resulting in single-particle mass spectra (positive mass spectra).

The obtained single-particle mass spectra were tested by using several statistical methods (K-means, principal component analysis (PCA), and ART-2a). The K-means algorithm was used to categorize the single-particle mass spectra because it had the best performance. The optimal number of classes or groups was determined by comparing the total separation variance (TSV) among them (i.e., similar mass spectra were grouped together). The tailing in the mass spectra could happen because no pulse signal was applied to the TOF-MS extraction plate. The *m/z* was adjusted to unit by rounding off, and the peak intensity was normalized before being used for the K-means algorithm.

Various polystyrene latex (PSL) particles with different sizes (125 nm, 300 nm, 700 nm, and 1000 nm) and inorganic particles with different chemical compositions were produced by using a constant atomizer (DS-A103, Dongsung Industry Inc., Hwaseong-si, Korea), where solutions were aerosolized and dried before being measured with the SPMS. Solutions for NaCl, KCl, FeSO_4_, CaCO_3_, and Al_2_O_3_, an internal mixture of NaCl and KCl, and an internal mixture of NaCl, KCl, and MgCl2 in deionized (DI) water were also prepared for the atomizer. All chemicals were purchased from Sigma-Aldrich, St. Louis, MO, USA.

**Figure 2:**
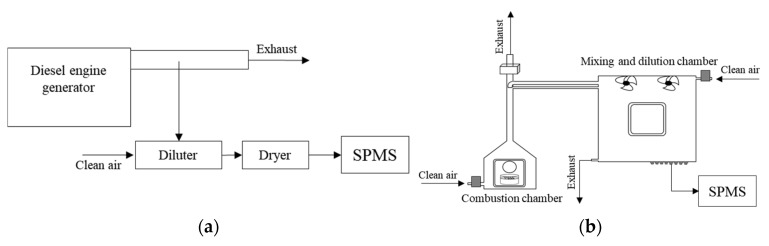

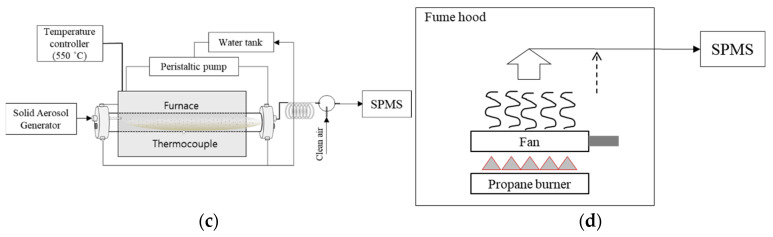
A schematic of various systems for generating combustion aerosols ((**a**) diesel engine, (**b**) biomass burning, (**c**) coal burning, and (**d**) meat burning).

Figure 2 shows various systems for generating combustion aerosols (diesel engine, biomass burning, coal burning, and meat burning). The particle generation systems were described in detail in our previous papers [33,34,35]. Briefly, diesel engine exhaust particles were produced from a diesel generator (192FC, Hi-earns, Changzhou, China), as shown in Figure 2a. The diesel generator (498 cc) was operated in idle mode at 3000 rpm. The tailpipe of the generator was directly exposed to the atmosphere, and an aerosol flow including diesel engine exhaust particles was sampled near the tailpipe. Then, aerosol flow with a flow rate of 1 L/m was mixed with particle-free air with a flow rate of 100 L/m to achieve dilution and cooling. The dilution ratio was around 100. For the generation of biomass burning particles, a biomass-burning chamber was constructed, as shown in Figure 2b. It consists of a combustion stove (0.8 m × 0.8 m × 0.8 m) and a dilution chamber (1.5 m × 1.5 m × 1.5 m). Approximately 25 g of rice straw, which was collected in an agricultural area of South Korea (Yeosu), was burned in the combustion stove. The rice straw is a major type of agricultural waste in South Korea and is usually burned intensively on farmland after harvest [36,37,38], causing severe local haze events. Coal combustion particles were produced by a coal-burning system (see Figure 2c) comprising a high-temperature tube furnace (Lindberg/Blue M, HTF55322C, Thermo Electron Corp., Franklin, MA, USA) and a dilution chamber. Bituminous coal was obtained from a coal power plant in South Korea (Korea South Power, Busan, Korea), and it was pulverized and screened (<75 μm) before being burned. The pulverized coal was fed into the tube furnace, where it was burned at a temperature of 550 °C. Then, coal burning particles were diluted approximately 100 times prior to the measurement. Meat (pork) cooking aerosols were produced by burning or grilling a piece of pork (belly part) with a propane burner, as shown in Figure 2d. Pork containing a high percentage of fat, which is burned on a pan, is a popular food in South Korea. Most of the smoke was exhausted into the fume hood, and an aerosol flow was sampled and mixed with dilution air before being measured with the SPMS. A series of diffusion dryers packed with silica gel and denuders (custom made) were installed in the aerosol flow line before being measured with the SPMS to remove water and volatile organic vapors from the aerosol flow. The scanning mobility particle sizer (SMPS) consisting of a differential mobility analyzer (3080, TSI, Shoreview, MN, USA) and a condensation particle counter (3788, TSI, USA) was used to determine the number size distribution of the generated particles.

## 3. Results and Discussion

A theoretical evaluation of the newly constructed aerodynamic lens system to focus particles into the center in the aerosol flow was conducted [28,32]. Ammonium sulfate particles were used as test aerosols. The transmission efficiency of the ammonium sulfate particles in a size range of 30 nm–10 µm was found to be higher than 90%. The particle’s terminal velocity was 224–322 m/s after passing through the aerodynamic lens. The focused particle beam diameter was less than 3 mm for particles with a diameter larger than 30 nm [29].

**Figure 3:**
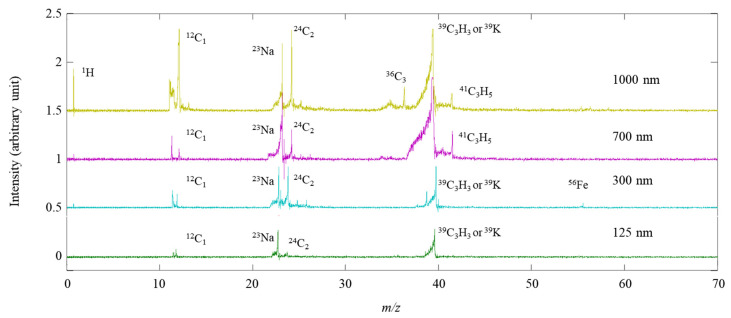
Single-particle mass spectra of PSL particles with different sizes (125 nm, 300 nm, 700 nm, and 1000 nm).

The single-particle mass spectra of PSL particles with various sizes (125 nm, 300 nm, 700 nm, and 1000 nm) are shown in Figure 3. Major peaks (C_1_ (*m/z* = 12) and C_2_ (*m/z* = 24) were identified for the PSL particles. Moreover, the Na^+^ (*m/z* = 23) and K^+^ (*m/z* = 39) peaks in the mass spectra were observed, which could originate from impurities in the PSL solution. The impurities of Na and K should become dominant when the partial ionization of particles by laser is strong. The Fe^+^ (*m/z* = 56) may originate from the electrodes under laser irradiation. The PSL particles were used to show that the SPMS is able to detect such particles of different sizes, and the accurate chemical signatures of PSL particles are not the focus of this research. All peak areas in the single-particle mass spectrum were summed to determine the total peak area, which increased as the particle size increased. However, the relationship was not as strong as in previous studies, suggesting that the complete ionization of particles was not achieved with less laser power intensity than that used in the previous studies [8,27].

**Figure 4:**
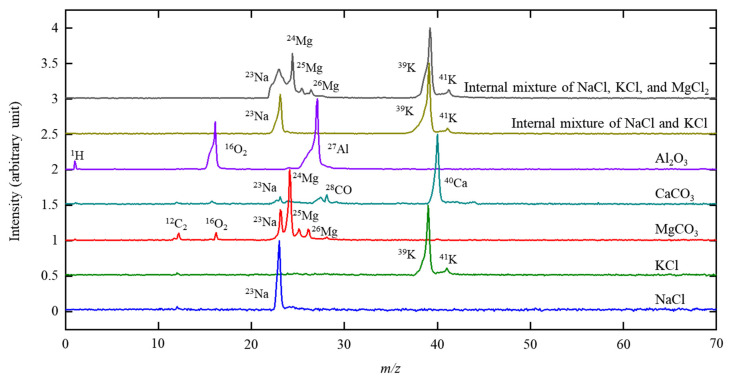
Single-particle mass spectra of NaCl, KCl, MgCO_3_, CaCO_3_, and Al_2_O_3_ particles; an internal mixture of NaCl and KCl; and an internal mixture of NaCl, KCl, and MgCl_2_.

The SPMS was also tested by using NaCl, KCl, MgCO_3_, CaCO_3_, and Al_2_O_3_ particles; an internal mixture of NaCl and KCl; and an internal mixture of NaCl, KCl, and MgCl_2_. Their single-particle mass spectra are shown in Figure 4. Major elements in these particles were clearly detected by the SPMS. The theoretical mass-to-charge ratios (*m/z*) for chemical elements agree well with the observed *m/z* values (y = 1.056 x − 0.7302 and r = 0.9994, where y is the observed *m/z*, x is the theoretical *m/z*, and r is the correlation coefficient). The Cl element was not detected under the current SPMS condition. Cl has often been detected in negative mass spectra [39]. Note that only positive mass spectra were measured in this study. The internal mixture of NaCl and KCl clearly had Na and K signals in the single-particle mass spectrum. Additionally, the internal mixture of NaCl, KCl, and MgCl_2_ clearly had Na, K, and Mg signals in the single-particle mass spectra. Our data suggest that the SPMS could be useful to determine the mixing states (internal mixture versus external mixture) of particles with different sizes.

**Figure 5:**
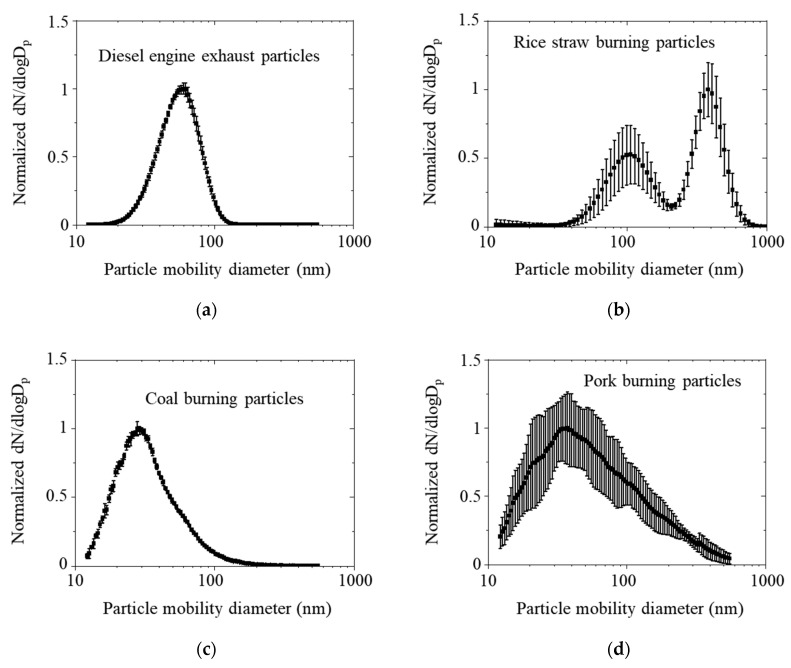
Particle number size distributions for various combustion aerosols ((**a**) diesel engine, (**b**) rice straw burning, (**c**) coal burning, and (**d**) pork burning).

Normalized particle number size distributions for various combustion aerosols are shown in Figure 5. Most of the particles were less than 1 µm (fine particles). The highest number of emissions was observed for the diesel engine exhaust particles with a mode diameter of 60 nm. The particles generated by rice straw burning had a bimodal distribution, and the first peak disappeared with the transition from flaming combustion to smoldering combustion [35]. The coal burning aerosols showed the smallest mode diameter, which was less than 30 nm (27.9 nm). The pork burning aerosols had a higher standard deviation in each size bin because they were generated by a more inhomogeneous burning process than the others.

**Figure 6:**
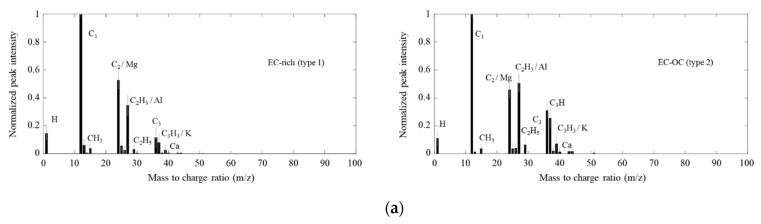

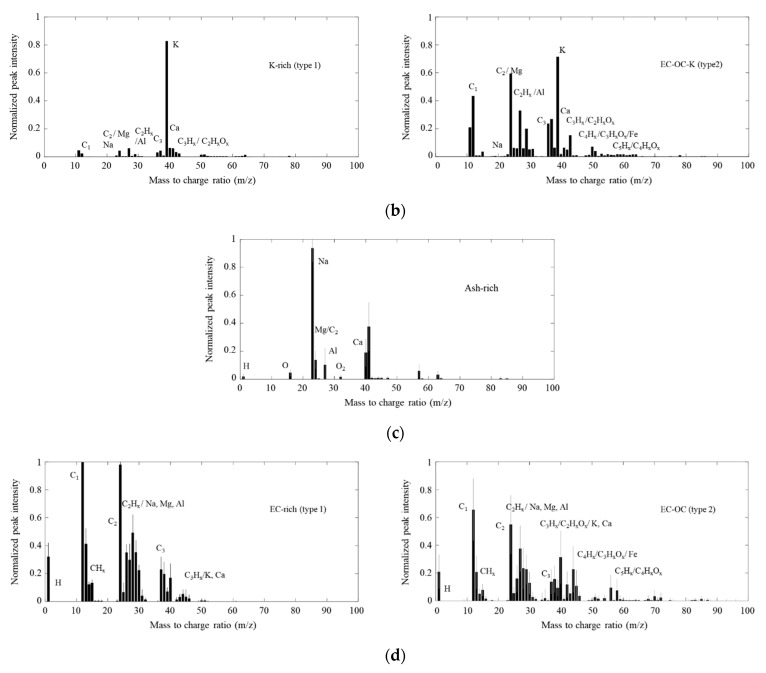
Single-particle mass spectra of (**a**) diesel engine exhaust particles, (**b**) rice straw burning particles, (**c**) coal burning particles, and (**d**) pork burning particles classified by the K-means algorithm.

The single-particle mass spectra obtained from the SPMS were classified by the K-means algorithm. Two major particle types were identified in diesel engine exhaust, as shown in Figure 6a. The EC-rich particles mostly consisted of elemental carbon (EC) (C_1_, C_2_, and C_3_) and a little organic carbon (OC) (CH_3_, C_2_H_3_, and C_2_H_5_), while the EC-OC particles included EC with a significant amount of OC (the C_2_H_3_ peak is higher than the C_2_ peak). Both particle types can be considered carbonaceous particles. In the diesel exhaust, the fraction of the EC-OC particles (74%) was higher than that of the EC-rich particles (26%). Diesel exhaust typically has higher particle number emissions and toxicity [33], which are a great concern for human health.

Two major particle types (K-rich and EC-OC-K particles) were observed among rice straw burning emissions, as shown in Figure 6b. K (*m/z* = 39), which is one of the essential elements for plants, was detected in all the single-particle mass spectra, supporting the notion that the K peak should be used as a chemical signature for biomass burning aerosols. The higher ionization efficiency of K compared to the other particles may also lead to a more significant K peak compared to other peaks. K-rich particles accounted for the highest fraction (68%), followed by EC-OC-K particles (5%). Biomass burning, including various wildfires and prescribed fires, is one of the largest sources of particulate matter in the world [40,41] and has significant effects on human health [42,43,44,45,46].

Only one particle type (ash-rich particles) was detected in coal burning emissions, as shown in Figure 6c. They mainly contained Na (*m/z* = 23), Mg (*m/z* = 24), Al (*m/z* = 27), and Ca (*m/z* = 40), consistent with earlier studies in which coal burning emissions contained a significant amount of minerals [47,48]. Pulverized coal combustion is typically used for the generation of electricity, accounting for around 40% of the world’s electricity [49]. In developing countries, coal is also used for household heating and cooking, which has adverse health effects on indoor residents [34,50,51,52].

Two types of particles (EC-rich and EC-OC particles) were observed among pork burning emissions, as shown in Figure 6d. The cooking process can contribute to high concentrations of indoor aerosols that affect human health [53]. EC-rich particles (36%) mainly consisted of C_1_, C_2_, and C_3_, as in the diesel exhaust particles but also comprised a small number of OC (e.g., CH_x_, C_2_H_x_, and C_3_H_x_). Note that the EC-rich particles produced from rice straw burning always had a K (*m/z* = 39) peak in the single-particle spectra, suggesting that the single-particle mass spectra of the EC-rich particles produced from the diesel engine, rice straw burning, and pork burning somewhat differed. For the EC-OC particles (62%), the OC peaks increased significantly. Organic compounds can include fatty acids in cooking aerosols [53,54].

The current SPMS measurement focused on the rapid classification of major particle types produced by various combustion sources rather than the accurate identification of organic compounds or speciation. Our data suggest that chemical signatures from various combustion aerosols can be identified. However, to successfully apply the SPMS technique for the rapid detection and classification of atmospheric aerosols on a single-particle basis, it is essential to obtain more single-particle mass spectra for other types of combustion aerosols (such as gasoline engine exhaust, pine wood burning, and fish cooking) and non-combustion aerosols (such as dust, sea-spray aerosols, secondary inorganic aerosols, and secondary organic aerosols). This will provide a useful method to accurately identify sources of fine particles and to better understand their effects on human health.

## 4. Conclusions

An SPMS with laser ionization was applied to detect and classify various fine particles (PSL particles with different sizes, inorganic particles and their mixtures, and combustion aerosols (diesel engine exhaust, rice straw burning, coal burning, and pork burning)) on a single-particle basis in real time. The major elements and mixing states (internal mixture versus external mixture) of the inorganic particles were successfully determined by the SPMS. Then, chemical signatures for the single-particle mass spectra of various combustion aerosols were obtained by SPMS measurements. EC-rich and EC-OC particles from diesel engine exhaust, K-rich and EC-OC-K particles from rice straw burning, ash-rich particles from coal burning, and EC-rich and EC-OC particles from pork burning were identified. The single-particle mass spectra of the EC or OC type of particles varied among different combustion sources. The EC-rich diesel engine exhaust particles mainly consisted of C_1_, C_2_, and C_3_, while EC-rich particles produced from rice straw burning always had a K peak in the single-particle spectra. The observed chemical signatures measured with the SPMS may be useful for identifying sources of atmospheric fine particles and for better understanding their effects on human health. However, more data on single-particle mass spectra for other types of combustion and non-combustion aerosols should be collected. In addition, the chemical signatures of fine particles detected by the SPMS may be used as an important parameter to infer their toxicity and effects on human health.

## Data Availability

The data presented in this study are available on request from the corresponding author.
